# The expression of FLNA and CLU in PBMCs as a novel screening marker for hepatocellular carcinoma

**DOI:** 10.1038/s41598-021-94330-1

**Published:** 2021-07-21

**Authors:** Rathasapa Patarat, Shoji Riku, Pattapon Kunadirek, Natthaya Chuaypen, Pisit Tangkijvanich, Apiwat Mutirangura, Charoenchai Puttipanyalears

**Affiliations:** 1 Center of Excellence in Molecular Genetics of Cancer and Human Diseases, Department of Anatomy, Faculty of Medicine, Chulalongkorn University, Bangkok, Thailand; 2 Tokyo Medical and Dental University, Tokyo, Japan; 3 Center of Excellence in Hepatitis and Liver Cancer, Faculty of Medicine, Department of Biochemistry, Chulalongkorn University, Bangkok, Thailand; 4 Department of Biochemistry, Chulalongkorn University, Bangkok, Thailand; 5 Department of Anatomy, Faculty of Medicine, Chulalongkorn University, 1873 Rama IV Road, Pathumwan, Bangkok, 10330 Thailand

**Keywords:** Cancer screening, Oncogenes, Hepatocellular carcinoma, Cancer microenvironment, Tumour biomarkers, Immunosurveillance

## Abstract

Early detection improves survival and increases curative probability in hepatocellular carcinoma (HCC). Peripheral blood mononuclear cells (PBMCs) can provide an inexpensive, less-invasive and highly accurate method. The objective of this study is to find the potential marker for HCC screening, utilizing gene expression of the PBMCs. Data from the NCBI GEO database of gene expression in HCC patients and healthy donor's PBMCs was collected. As a result, GSE 49515 and GSE 58208 were found. Using both, a statistical significance test was conducted in each gene expression of each data set which resulted in 187 genes. We randomized three selected genes (FLNA, CAP1, and CLU) from the significant *p*-value group (*p*-values < 0.001). Then, a total of 76 healthy donors, 153 HCC, 20 hepatic fibrosis, 20 non-alcoholic fatty liver were collected. Quantitative RT-PCR (qRT-PCR) was performed in cDNA from all blood samples from the qRT-PCR, The Cycle threshold (Ct) value of FLNA, CLU, CAP1 of HCC group (28.47 ± 4.43, 28.01 ± 3.75, 29.64 ± 3.90) were lower than healthy group (34.23 ± 3.54, 32.90 ± 4.15, 32.18 ± 5.02) (*p*-values < 0.0001). The accuracy, sensitivity and specificity of these genes as a screening tool were: FLNA (80.8%, 88.0%, 65.8%), CLU (63.4%, 93.3%, 31.3%), CAP1 (67.2%, 83.3%, 39.1%). The tests were performed in two and three gene combinations. Results demonstrated high accuracy of 86.2%, sensitivity of 85% and specificity of 88.4% in the FLNA and CLU combination. Furthermore, after analyzed using hepatic fibrosis and non-alcoholic fatty liver as a control, the FLNA and CLU combination is shown to have accuracy of 76.9%, sensitivity of 77.6% and specificity of 75%. Also, we founded that our gene combination performs better than the current gold standard for HCC screening. We concluded that FLNA and CLU combination have high potential for being HCC novel markers. Combined with current tumor markers, further research of the gene’s expression might help identify more potential markers and improve diagnosis methods.

## Introduction

Hepatocellular carcinoma (HCC) is the fourth most common cause of cancer-related mortality worldwide^1^. Annually, liver cancer accounts for approximately 840,000 new cases and 780,000 death cases^2^. In Thailand, HCC tends to be diagnosed late and until it becomes worse, for example, 75% of patients diagnosed as HCC have already reached stage B or C in Barcelona Clinic Liver Cancer staging, and 25% of the patients were asymptomatic^3^.

Primary screening for HCC is mainly conducted either by Alpha-Fetoprotein (AFP) level from blood sample or by ultrasound imaging. If an abnormality is found, a contrast-enhanced multiphase Computerized Tomography or Magnetic Resonance Imaging study would be done^4^. However, the AFP blood test produces a wide variation of results with sensitivity ranging from 32 to 79.5% and specificity ranging from 29.4 to 98.5%^5–7^. Ultrasound is also problematic because it is operator dependent. The results may vary from each operator thus, reducing its capability. Ultrasound test sensitivity for the detection of HCC ranges from 29 to 100%, whereas its specificity ranges from 94 to 100%. This means both AFP and ultrasound performance as screening/diagnosis markers are not very satisfactory^8^.

**Figure 1:**
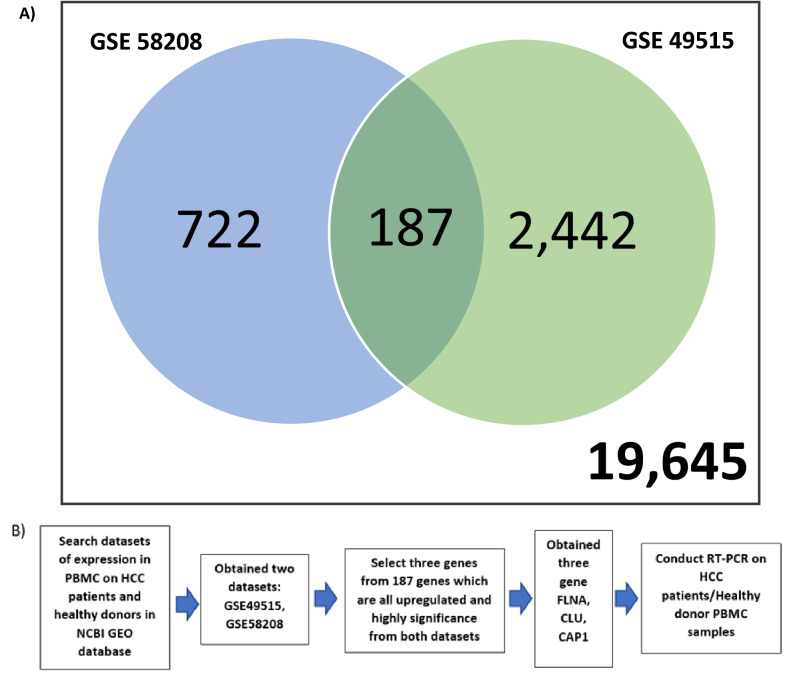
Summary of the experiment. (**a**) Venn diagram based on the overlapping number of upregulated genes between two microarray data extracted from Gene Expression Omnibus (GEO) data repository. (**b**) A schematic design flow of the experiment.

Recently, there are multiple studies regarding the change in gene expression in circulating white blood cells of the cancer patient^9–11^. Also, there are experiments utilizing the gene expression on peripheral white blood cells to detect these cancer’s influences^12–16^. Therefore, an invention of markers that are inexpensive, simple, less-invasive and highly accurate can be expected. We focused on the usability of peripheral white blood cells (WBCs), especially peripheral blood mononuclear cells (PBMCs) as a biomarker for liver cancers^17^. The objective of this study is to find the high-performance novel markers for HCC screening, utilizing gene expression of PBMCs (Fig. 1b).

## Results

### Data summary

#### Bioinformatic data of the samples

Results of the CU-DREAM program showed a significant *p*-value in the comparison between GSE49515^18^ and GSE58208^19^ (*p*-value = 9.91 × 10^−19^, Odd ratio = 2.08, upper 95%CI = 2.46 and lower 95%CI = 1.76). All 187 upregulated genes were classified to identify the highest significant *p*-value. Then, three genes with high significant *p*-values including CLU (*p*-value = 8.16 × 10^−5^), FLNA (*p*-value = 3.35 × 10^−5^), and CAP1 (*p*-value = 2.84 × 10^−7^) were selected and applied to observe the gene expression in this study (Fig. 1a, Table 1) .

**Table 1:** Results from the bioinformatics approach (CU-DREAM program) demonstrated the 187 upregulated genes (*p*-value = 9.91 × 10^−19^, Odd ratio = 2.08, upper 95%CI = 2.46 and lower 95%CI = 1.76) from the intersection of GSE58208 and GSE49515 dataset (level of significance *p-value < 0.001).

GSE49515	GSE58208		
	Upregulated	Not upregulated	Total
Upregulated	187	2442	2629
Not Upregulated	722	19,645	20,367
Total	909	22,087	22,996

#### Characteristics of participants

Age (Mean ± SD) of HCC group was 58.93 ± 9.99, Healthy donor group was 48.32 ± 5.16, hepatic fibrosis (HF) group was 57.85 ± 8.92, and non-alcoholic fatty liver (NAFL) group was 57.50 ± 8.31. Gender data shows that our samples had more males than females (Table 2); The HCC group has 124 males to 29 females, the Healthy group has 45 males to 31 females, the HF group has 16 males to 4 females, and the NAFL group has 18 males to 2 females. The staging of HCC from the cancer group showed 12 samples in stage 0, 54 samples in stage A, 61 samples in stage B, 26 samples in stage C, and no samples in stage D according to the BCLC staging system.

**Table 2:** Demographic data of samples. HCC (N = 153), HF (N = 20), NAFL (N = 20), Healthy Donor (N = 76).

Data	HCC	HF	NAFL	Healthy
**Age**				
Mean ± SD	58.93 ± 9.99	57.85 ± 8.92	57.50 ± 8.31	48.32 ± 5.16
Median	58	57.5	57.5	48
**Gender**				
Male	124	16	18	45
Female	29	4	2	31
**Tumor size (maximum)**				
< 2 cm	37	–	–	–
2–3 cm	29	–	–	–
> 3 cm	87	–	–	–
**Number of tumors**				
1	66	–	–	–
2	30	–	–	–
3	17	–	–	–
> 3	40	–	–	–
**Laboratory test result (mean ± SD)**				
Hemoglobin (g/dL)	12.36 ± 1.95	14.74 ± 1.03	14.86 ± 0.91	–
Hematocrit (%)	37.60 ± 5.02	43.28 ± 1.74	43.65 ± 1.67	–
Platelet count (10^9^/L)	164.08 ± 91.58	201.3 ± 58.02	248.6 ± 62.24	–
WBC count (10^9^/L)	6.21 ± 3.59	3.96 ± 2.39	6.76 ± 1.65	–
Creatinine (mg/dL)	0.90 ± 0.52	1.01 ± 0.43	1.07 ± 0.55	–
Total Bilirubin (mg/dL)	1.00 ± 0.73	1.14 ± 0.50	0.57 ± 0.17	–
Direct Bilirubin (mg/dL)	0.48 ± 0.34	0.42 ± 0.15	0.23 ± 0.08	–
Serum albumin (g/dL)	3.73 ± 2.52	4.30 ± 0.37	4.34 ± 0.24	–
Aspartate aminotransferase (IU/L)	67.68 ± 44.95	39.71 ± 25.38	28.45 ± 10.47	–
Alanine aminotransferase (IU/L)	53.73 ± 38.62	46.94 ± 35.01	34.50 ± 18.33	–
Alkaline phosphatase (IU/L)	133.41 ± 68.56	78.00 ± 15.71	56.38 ± 8.40	–
INR	1.20 ± 0.23	1.66 ± 0.62	1.00 ± 0.22	–
Alpha fetoprotein (ng/mL)	13,251.54 ± 69,742.37	10.24 ± 14.75	2.20 ± 0.67	–
**Child–Pugh score**				
A	80	20	20	–
B	16	–	–	–
C	57	–	–	–
**HCC stage (BCLC staging)**				
0	12	–	–	–
A	54	–	–	–
B	61	–	–	–
C	26	–	–	–
D	0	–	–	–
**Present of Cirrhosis**				
Yes	123	1	2	–
No	30	19	18	–

The laboratory result of The HCC group show that more than half of the HCC patient has Child–Pugh class A (80 from 153) while class C are lesser in number (57 from 153) and class B has the least number (16 from 153). Also, the majority of the HCC samples have cirrhosis (120 from 153). The Blood cell count is notably within the normal range. Hemoglobin is 12.36 ± 1.95 (g/dL), Hematocrit (%) is 37.60 ± 5.02, Platelet count (10^9^/L) is 164.08 ± 91.58, and WBC count (10^9^/L) is 6.21 ± 3.59. The liver function test is predictively increased to that of chronic liver disease. Aspartate aminotransferase (IU/L) level is 67.68 ± 44.95, Alanine aminotransferase (IU/L) level is 53.73 ± 38.62, Alkaline phosphatase level (IU/L) is 133.41 ± 68.56. The serum alpha fetoprotein level is also increased (13,251.54 ± 69,742.37 ng/mL). Additionally, the HF and the NAFL groups’ laboratory result shown that both are within normal range. Sample from both HF and NAFL group are belong only to class A from Child–Pugh classification system (Table 2).

#### Quantitative Real-time PCR analysis with 2^−ΔΔCt^ calculation

**Figure 2:**
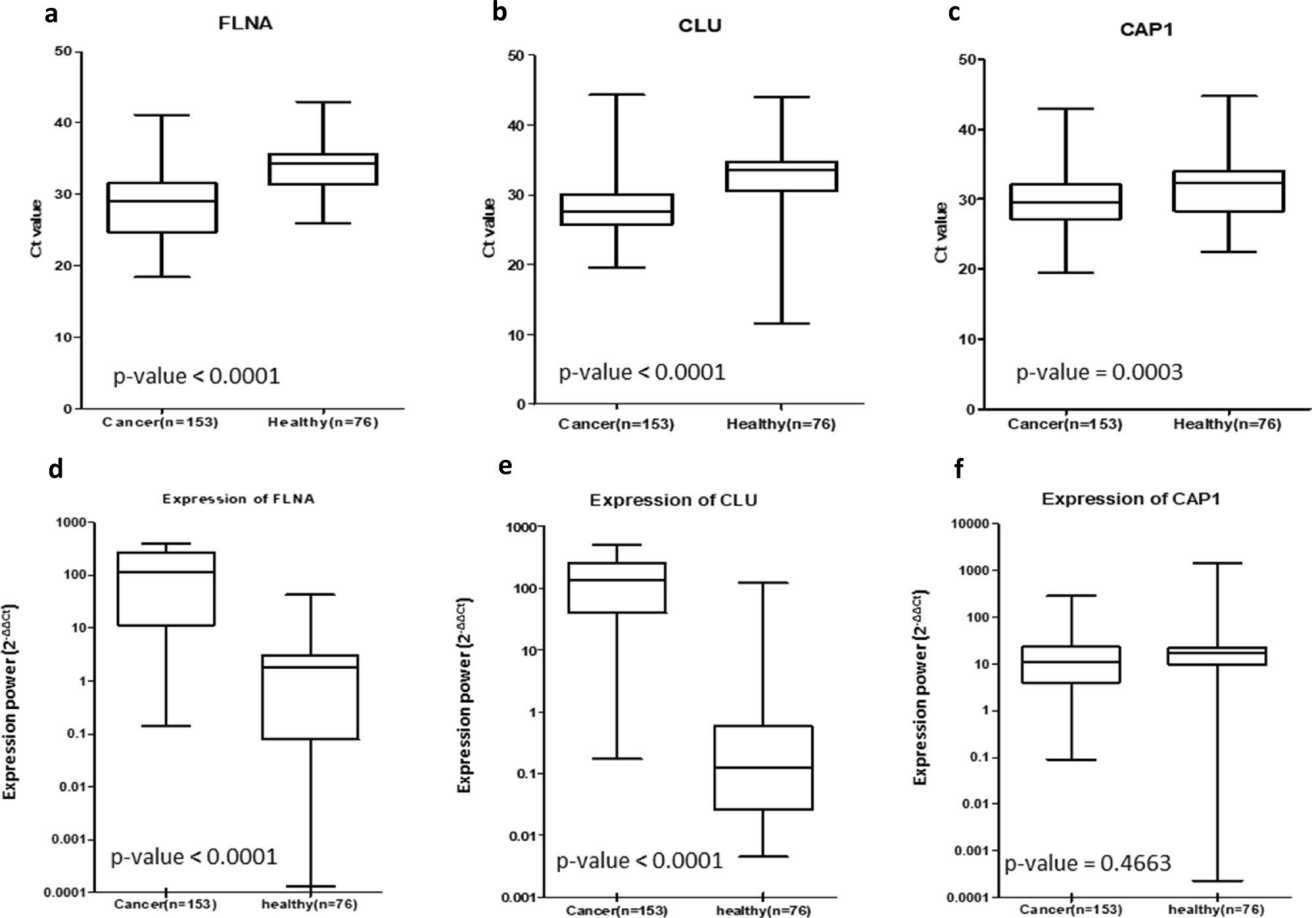
Ct values and expression (2^−ΔΔCt^) of each gene compare to the housekeeping gene (GAPDH) within Healthy Donor group and HCC cancer group, shown in boxplot (Mean ± SD); (**a)** Ct value of FLNA gene, (**b)** Ct value of CLU gene, (**c)** Ct value of CAP1 gene, (**d)** Expression of FLNA gene, (**e)** Expression of CLU gene, (**f)** Expression of CAP1 gene. The Ct values in HCC group were significantly lower than the healthy group (*p*-value < 0.0001 in FLNA, CLU and *p*-value = 0.0003 in CAP1). The expression of FLNA, CLU, CAP1 was increase in the HCC group when compare with the healthy group (*p*-value < 0.0001 in FLNA and CLU and *p*-value = 0.4663).

In the HCC group, The Ct value was 28.47 ± 4.43 for FLNA, 28.01 ± 3.75 for CLU and 29.64 ± 3.90 for CAP1. The level of Ct value in the healthy group was 34.23 ± 3.54 for FLNA, 32.90 ± 4.15 for CLU and 32.18 ± 5.02 for CAP1. The Ct value of HF group was 30.75 ± 3.90 for FLNA, 32.14 ± 0.78 for CLU and 30.01 ± 4.94 for CAP1. The Ct value of NAFL group was 30.46 ± 3.45 for FLNA, 32.13 ± 0.80 for CLU and 28.98 ± 3.30 for CAP1 (Table 3). These results demonstrated that the Ct values in HCC group were significantly lower than the healthy group, (*p*-value < 0.0001 in FLNA, CLU and *p*-value = 0.0003 in CAP1) (Fig. 2).

**Table 3:** Result data after analysis. All gene expression is result from Real time – PCR and represent by cyclic threshold (Ct) (mean ± SD) then analyzed using either ANOVA or t-test.

Result data	HCC	HF	NAFL	Healthy	*p*-value
**Overall expression (Ct) (mean ± SD)**					
FLNA	28.47 ± 4.43	30.75 ± 3.90	30.46 ± 3.45	34.23 ± 3.54	< 0.0001
CLU	28.01 ± 3.75	32.14 ± 0.78	32.13 ± 0.80	32.90 ± 4.15	< 0.0001
CAP1	29.64 ± 3.90	30.01 ± 4.94	28.98 ± 3.30	32.18 ± 5.02	0.0003
**Early cancer (stage 0, A) and control (Ct)**					
FLNA	28.18 ± 4.00	30.75 ± 3.90	30.46 ± 3.45	34.23 ± 3.54	< 0.0001
CLU	27.70 ± 2.89	32.14 ± 0.78	32.13 ± 0.80	32.90 ± 4.15	< 0.0001
CAP1	29.15 ± 3.27	30.01 ± 4.94	28.98 ± 3.30	32.18 ± 5.02	0.0007
**FLNA gene between cancer stage (Ct)**					
0	27.97 ± 4.19				0.7338
A	28.23 ± 3.99				
B	28.78 ± 4.32				
C	27.69 ± 5.47				
D	–				
**CLU gene between cancer stage (Ct)**					
0	28.75 ± 3.11				0.0773
A	27.48 ± 2.84				
B	28.63 ± 4.24				
C	26.23 ± 3.27				
D	–				
**CAP1 gene between cancer stage (Ct)**					
0	29.32 ± 3.16				0.3943
A	29.12 ± 3.33				
B	30.22 ± 4.24				
C	28.71 ± 3.56				
D	–

Moreover, when we utilized either HF group or NAFL group or the combined HF and NAFL group as the control and compare each gene expression against HCC group, we found that only CLU gene is shown to express significantly difference from other genes. The significant different of each gene expression when pairing between HCC group and HF group are *p*-value = 0.08 for FLNA gene, *p*-value < 0.0001 for CLU gene, *p*-value = 0.64 for CAP1 gene. The significant different of each gene expression when pairing between HCC group and NAFL group are *p*-value = 0.06 for FLNA gene, *p*-value < 0.0001 for CLU gene, *p*-value = 0.32 for CAP1 gene. The significant different of each gene expression when pairing between HCC group and HF group with NAFL group are *p*-value = 0.013 for FLNA gene, *p*-value < 0.0001 for CLU gene, *p*-value = 0.34 for CAP1 gene. Also, there is no statistically significant difference of each gene expression between HF group and NAFL group (Table 4).

**Table 4:** Additional analysis. The table shown the statistically significance (*p*-value) of all gene expression when pairing between HCC group, HF group, and NAFL group (all using t-test).

Gene	Pairing			
	HCC & HF	HCC & NAFL	HF & NAFL	HCC & NF + NAFL
FLNA	0.08	0.06	0.99	0.013
CLU	< 0.0001	< 0.0001	0.92	< 0.0001
CAP1	0.64	0.32	0.82	0.34

Furthermore, we used the 2^−ΔΔCt^ method to calculate expression power compared to the house keeping gene from both HCC and healthy groups. We found that within the HCC group FLNA, CLU, CAP1 gene expressed (Median) 112.7 folds, 134.2 folds, 11.3 folds to the house keeping gene expression, respectively while the Healthy group expressed 1.9 folds, 0.1-fold, 17.1 folds to the house keeping gene, respectively with *p*-values of < 0.0001, < 0.0001, 0.4663, respectively (Fig. 2).

#### The performance of genes as a screening test

**Figure 3:**
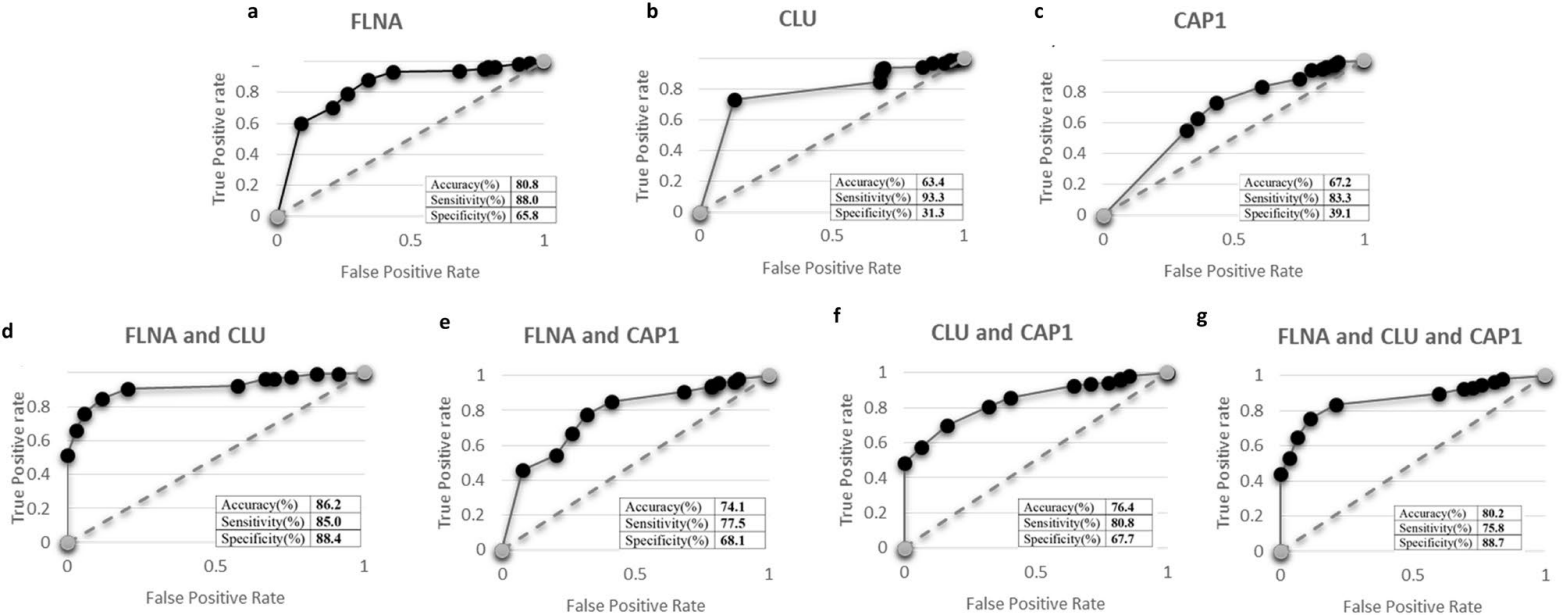
The ROC graph and the result of accuracy, sensitivity, specificity in each combination including; (**a)** FLNA, (**b)** CLU, (**c)** CAP1, (**d)** FLNA and CLU, (**e)** FLNA and CAP1, (**f)** CLU and CAP1, (**g)** FLNA and CLU and CAP1. The combination of FLNA and CLU has the greatest discriminate capacity than other tests (86.2%accuracy, 85.0%sensitivity, 88.4%specificity).

The performance of results (Fig. 3) are reported hereafter. When using the cut-off value of Ct value < 33, the results of accuracy, sensitivity and specificity were FLNA (80.8% accuracy, 88.0% sensitivity, 65.8% specificity) (Fig. 3a), CLU (63.4% accuracy, 93.3% sensitivity, 31.3% specificity) (Fig. 3b) and CAP1 (67.2% accuracy, 83.3% sensitivity, 39.1% specificity) (Fig. 3c). Then, the two and three-gene combinations were performed. The results showed that the combination of FLNA & CLU (86.2% accuracy, 85.0% sensitivity, 88.4% specificity) (Fig. 3d) demonstrated higher proficiency than the combination of FLNA & CAP1 (74.1% accuracy, 77.5% sensitivity, 68.1% specificity) (Fig. 3e), CLU & CAP1 (76.4% accuracy, 80.8% sensitivity, 67.7% specificity) (Fig. 3f). However, the three-gene combination could not affect the efficiency of the test (80.2% accuracy, 75.8% sensitivity, 88.7% specificity) (Fig. 3g). From all the ROC graphs (Fig. 3) we showed that the combination of FLNA & CLU has the greatest discriminate capacity than the other tests.

**Figure 4:**
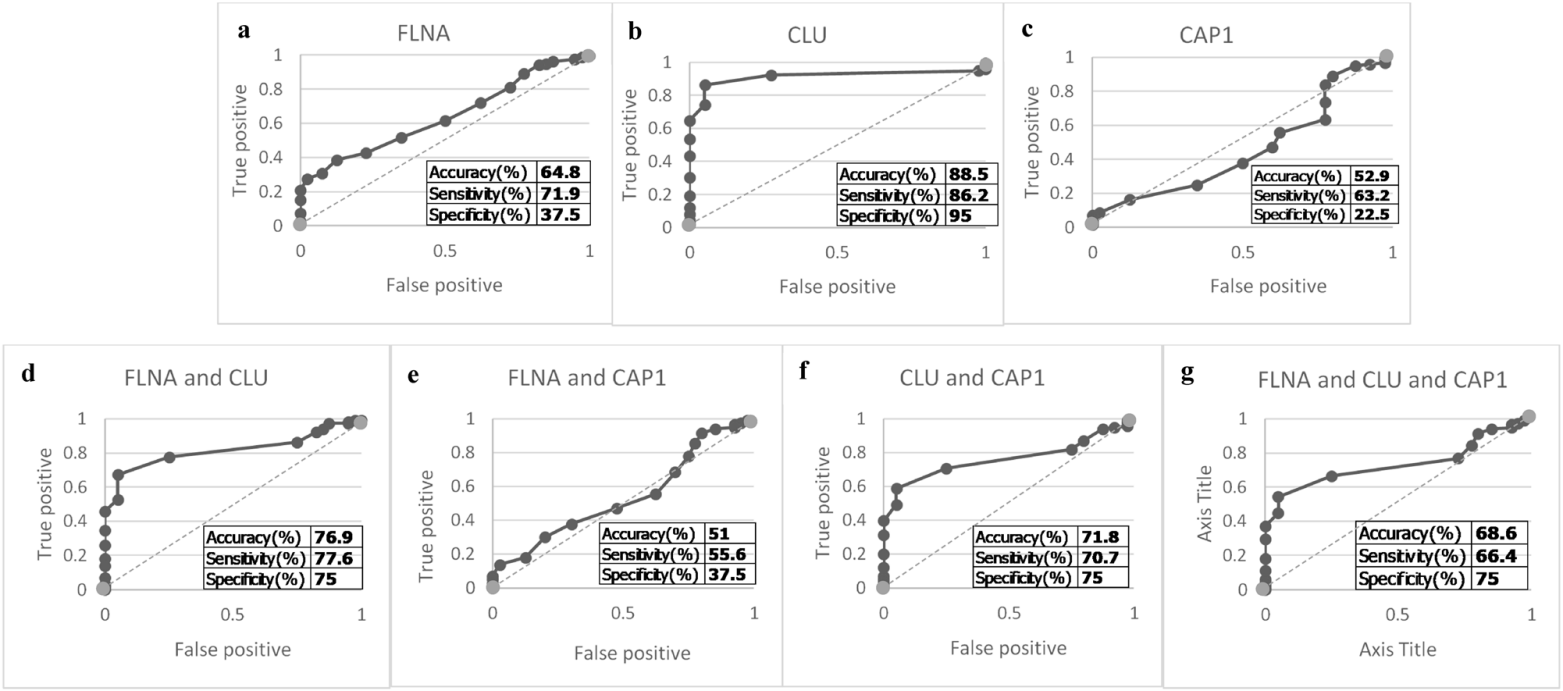
The ROC graph and the result of accuracy, sensitivity, specificity in each combination including; (**a)** FLNA, (**b)** CLU, (**c)** CAP1, (**d)** FLNA and CLU, (**e)** FLNA and CAP1, (**f)** CLU and CAP1, (**g)** FLNA and CLU and CAP1.This analysis use samples from the hepatic fibrosis group and the non-alcoholic fatty liver group as a control.

The performance of the genes when using sample from HF group with NAFL group are as follow. When using the cut-off value of Ct value < 31 the results of accuracy, sensitivity and specificity were FLNA (64.8% accuracy, 71.9% sensitivity, 37.5% specificity) (Fig. 4a), CLU (88.5% accuracy, 86.2% sensitivity, 95% specificity) (Fig. 4b) and CAP1 (52.9% accuracy, 63.2% sensitivity, 22.5% specificity) (Fig. 4c), FLNA & CLU (76.9% accuracy, 77.6% sensitivity, 75% specificity) (Fig. 4d), FLNA & CAP1 (51% accuracy, 55.6% sensitivity, 37.5% specificity) (Fig. 4e), CLU & CAP1 (71.8% accuracy, 70.7% sensitivity, 75% specificity) (Fig. 4f), and the three-gene combination (68.6% accuracy, 66.4% sensitivity, 75% specificity) (Fig. 4g). From all the ROC graphs (Fig. 4).

In addition, the performance of the genes to detect the early liver cancer stage is also analyzed. We found that all three genes can distinguish between the healthy group and the early HCC group (Table 3). However, all three genes show no statistically significant distinction between liver cancer stages (Table 3).

Furthermore, we test the early HCC group (stage 0, A of BCLC staging system) (66 subjects) with both the current gold standard for HCC screening (AFP and ultrasonography) and our gene combination (FLNA and CLU). We found that our gene combination is superior in identifying the early HCC than the gold standard. While the gold standard has an accuracy of 30.3% (20 of 66), our gene combination has accuracy of 69.7% (46 of 66) with *p*-value < 0.0001. This reinforces the capability of our gene combination as a novel screening marker for HCC.

## Discussion

FLNA and CLU gene combination might be a prospective marker for HCC. We demonstrated that the PBMCs are affected by the HCC and the result contained the upregulated FLNA and CLU gene (86.2% accuracy, 85.0% sensitivity, 88.4% specificity) which possess high performance as novel screening markers for discriminating the presence or absence of HCC in patients. Furthermore, both genes shown to be able to separate early cancer patient and healthy people. Moreover, this test only requires peripheral blood for testing e.g., AFP and is considered one of the least invasive types of testing available. Furthermore, this test is operator independent, unlike ultrasound which require years of training and hours of procedures to be able to produce reliable results.

From the result of our initial analysis of the microarray data, we found that there are differences in gene expressions of the PBMCs between HCC patients and normal people (Fig. 1a, Table 1). The genes that significantly upregulate are involved in the immunotolerance process, anti-apoptosis, and pro-proliferative pathways. The mechanisms behind these modifications are still unknown but could be theoretically divided into three processes.

The first process is by various cytokines released from cancer cells or the immune cell responding to the cancer cells. The cytokines and chemokines are known to be involved in the interaction between malignant cells and immune cells^20–23^. Mostly, in the roles of chemotaxis, pro-angiogenesis, and inflammatory response. Recent studies show that the cancer cells are responsible for the changes in the release of the cytokines from PBMCs and those cytokines are also associated with tumor growth and invasion^24^.

The second process is by direct cell to cell interaction between the cancer cells and immune cells. Cancer cells and immune cells have a predator–prey-like relationship. If the immune cells recognize the cancer cell, the immune cell will initiate the apoptotic process of the recognized cancer cell^25^. But, if the immune cells fail to recognize the cancer cell, it will continue to grow unimpeded. This process is governed by multiple receptors and ligands such as major histocompatibility complex (MHC), program death ligand 1 or 2 (PD-L1 or PD-L2), CD80, CD86, CD28, cytotoxic T-lymphocytes-associated protein 4 (CTLA-4), T-cell receptor (TCR), and many others^26,27^. The malignant cell could disrupt these processes by genetic mutations. The result of the mutation could lead to the transmutations or the removal of the receptor/ligand/key protein in the processes interrupting the immune cell response. Furthermore, the alteration in these cell–cell signals might lead to the change in WBCs itself. A recent study shows that there is a dynamic relationship between the cancer cells and peripheral immune cell phenotype^28^. If the cancer cell has over-expressed PD-L1 it will lead immune cells’ dysfunction and also lead to the reduction in the number of circulating immune cells because the proliferation of the immune cells is prohibited^29^. This suggests a connection between cell–cell interaction and immune cells gene expressions.

The third process is due to the shift in the body's physiology that causes the changes in gene expression of PBMCs. the third process is targeting the macro-environment of the cancer cells. This third process involves multiple origins such as the inherited genetic susceptibility, the socio-economic issues which lead to different lifestyles (e.g., Alcohol consumption, smoking, profession-related environment, etc.), and mental health issue (e.g., stress from both personal or from the society at large). These will result in changes in the internal mechanism of the body and could potentially cause the change in PBMCs^30–34^. Multiple studies report on the correlation between stress and immune responds^35^. Some studies report on alcohol consumption and immunosuppression. All of this supports the approach of the immune cell gene expression is tied to whole-body physiology status. However, the precise mechanisms responsible for these three processes are still eluding the scientists. Thus, further research into the component of these pathways may benefit both the laboratory and clinical fields. This not only includes many possible laboratory investigations but also a potential treatment for multiple cancer as well.

The relationship between HCC cells and each candidate gene does exist. There are reports on FLNA gene that it has a complex role on the cytoskeleton^18^. FLNA gene encodes Filamin A protein. This protein is a non-muscle actin filament cross-linking protein^36^. The most common role of this protein is for being a scaffolding molecule to regulate various cell functions. More than 90 types of protein are reported to bind to this protein, and many of those genes are responsible for cytoskeleton reorganizing^37^. Thus, making it involved in cell migration by prompting transcription factor SRF along with MLK1^38^. This act of triggering both genes result in the tumor undergoes local invasion and metastasis. It is also reported that elevated FLNA gene expression in the harvested HCC tissue could be a predictor of recurrence of HCC^39^ and could also be a marker for the progression of HCC^40^. Moreover, there is a report on the importance of Filamin A protein within the immune cell. Mainly, Filamin A is necessary for the activation of human T-cells^41^. Protein kinase C-θ (PKC-θ) which plays a major role in the early activation of T-cell is reported to require Filamin A protein to translocate itself within the cell. There is also an association between PKC-θ and Interleukin-2 (IL-2) production. IL-2 is a cytokine essential in the maturation and differentiation of the regulatory T-cell which responsible for immunotolerance. The PKC-θ is reported to target Activator protein 1 (AP-1), Nuclear Factor Kappa B (NF-κB), and nuclear factor of activated T-cells (NFAT) which are the transcription factors of the IL-2 gene. The suppression in Filamin A synthesis is reported to result in the reduction of IL-2 production. This predicts an outcome when the cancer cell is successfully evading the immune cell and induced immune tolerance to cancer cell lineage. The demand for regulatory T-cells is increased because the need to suppress the immune response of cancer cells is increased. This required more IL-2 production and PKC-θ activation. Thus, need more Filamin A synthesis which results in overexpress of FLNA gene in PBMCs. This suggests that FLNA gene expression in the immune cell might be linked to the survival of cancer cells. However, multiple studies also report on the tumor-suppressive ability of the Filamin A^42^. It suppresses tumor growth and reduces tumor invasion by inhibiting transcription of the oncogenes when it has transmigrated into the nucleus.

CLU gene encodes Clusterin protein. This protein is involved in the clearance of cell debris and apoptosis pathway^43^. Its main function is to be a chaperone molecule that helps prevent cells to undergoes apoptosis by preventing the pro-apoptotic protein to bind to the mitochondria^44^. It has been reported that Clusterin is involve in many tumorigeneses’ activity. The protein is shown in vitro to involved in cell survival and aggregation^45^, promoting metastasis of HCC^46,47^, protecting the HCC cells from endoplasmic reticulum stress induced apoptosis^48^, regulating the NF-κB pathway which controls the innate immune response of the cell, and affecting resistance to drugs (such as Sorafenib)^49,50^. Clusterin is also reported to play a role in immunotolerance to the autoimmune associated antigen. After antigen presenting cells (APCs) clear the apoptotic cells by phagocytosis, it will present the antigen derive from the apoptotic cells. Any antigen that got present this way led to an increase in immune tolerance to it. The APCs also produce immunoregulatory cytokine (such as Transforming Growth factor-beta (TGF-β), interleukin-10 (IL-10)) to suppress local inflammatory response and prevent the development of an autoimmune disease^51^. Without a clearance of apoptotic cells, it will undergo necrosis and trigger an inflammatory response from APCs which led to the different pathway of presenting the antigen. When an antigen got present via inflammatory response, it will be recognized as a foreign element and the immune system will produce antibodies to combat it. If the autoimmune associated antigen got recognized then it could cause the autoimmune disease. Clusterin is reported to enhance the apoptotic way of presenting the antigen within T-cell, APCs and also delayed the transformation of apoptotic cells to necrosis cells. This suggests the association between the cancer cell and Clusterin protein level of the immune cell. The cancer cells could undergo apoptosis by themselves from over-mutation or by other causes and the apoptotic cancer cells got phagocytosis by APCs but get recognized as self-antigen. This led to the immune tolerance to the cancer cells and increase cancer cells’ survival. Then, the survival cancer cell multiplied, and more undergoes apoptosis. This causes more apoptotic cell clearance and the need for Clusterin protein. Thus, could lead to the up-regulation of the CLU gene in PBMCs.

CAP1 gene encodes Cyclase-Associated Protein 1(CAP1 protein). A study of the CAP1 gene reported its involvement in the metastasis of hepatocellular carcinoma due to its relationship to the actin filaments turnover cycle and also hypothesized that the cancer cell invasion will be accelerated when the gene is over-expressed^52^. It is also thought to be involved in the localization of cell polarity and mRNA^53^. Another study has reported that CAP1 gene expression increases in other cancers, such as ovarian cancer, and is involved in cell proliferation^54^. Moreover, CAP1 protein plays a part in stimulating monocyte and cause local inflammation in human^55^. CAP1 protein is a receptor to the resistin protein. Resistin is a cytokine involved in chronic inflammatory diseases such as atherosclerosis and insulin resistance. When resistin bind to CAP1 protein in monocyte it will activate cAMP-dependent signaling pathway, PKA, and NF-kB. These cause the increase in the production of pro-inflammatory cytokines such as IL-6, TNFα, and IL-1β. Up-regulate CAP1 in monocyte enhance the resistin-induced activity and cause low-level inflammation to develop into chronic inflammation. Cancer is associated with chronic inflammation^56^. By repeatedly undergoes inflammation, the cells will accumulate both DNA damages and mutations which could cause the transmutation from normal cells to cancer cells. If low-level inflammation accumulates in the cancer cells region, it will promote cancer cells’ development. This suggests the association between CAP1 gene expression in PBMCs and the development of cancer.

It may be possible to think that cancer cells reshape the PBMC with various mechanism. An experiment was conducted in our laboratory in breast cancer cells which showed the actual “reshaped” by cancer cell was carried out, although the detailed mechanism is unknown. The same phenomenon seemed to be occurring also in the case of HCC. Future study to clarify the mechanism are needed.

The benefits of this study (qRT-PCR assay) are apparent. Currently, the diagnosis of HCC has been done with imaging tests (Ultrasound, CT, MRI), conventional markers like AFP, or biopsy. However, all of them have limitations: imaging tests are quite expensive and not very suitable for wide screening and some tests are operator dependent; conventional markers have unsatisfiable accuracy; the pathological diagnosis using biopsy is highly invasive. Our FLNA and CLU combination markers, on the contrary, can be attained by less invasive blood test, yield high performance, and could be done with lower cost. Additionally, our genes can differentiate between patient with HCC from the patient with predisposing condition such as hepatic fibrosis, and non-alcoholic fatty liver (Table 4) but the gene combination suffer a little reduction in the performances (Fig. 4). Our markers could be expected to contribute in both screening and diagnosis of HCC in future clinical application.

The limitations of this study include the absence of in vitro investigation of this experiment. This is because the researchers have discussed and theorized on the processes behind the alteration in PBMCs’ gene expression to be a multifactorial process and determine that a co-culture between HCC cell line and PBMCs is not necessarily required. This is because the co-culture could only reflect one of the three processes that led to the change in gene expression and such a test could only produce a short-term interaction between the cancer cell and white blood cell. Moreover, the HCC cell line and PBMCs will be coming from a different source which might lead to the different reactions from the immune cells and result in the different genes being up-regulated. Therefore, the researcher judged that this experiment did not require an in vitro investigation of co-culture between HCC cells and PBMCs. Even though, this study did compare samples of patients with predisposing factor such as HF and NAFL, the experiment still not include other malignant disease from the nearby organ (such as cholangiocarcinoma, pancreatic cancer, etc.) that may share similar genes expression. In which case, the accuracy may be affected in clinical application. Therefore, in the future, investigating whether elevated expression of the gene is specific to patients only with HCC is warranted.

## Methods

### Method statement

All research was performed in accordance with relevant guidelines and regulations.

### Bioinformatics analysis

In this study, we recruited a bioinformatics approach to narrow the candidate genes for potential markers. In the Gene Expression Omnibus (GEO) repository of NCBI, microarray analysis results submitted by worldwide researchers are made available. From the NCBI database, data sets of gene expressions in PBMC were searched. Search terms were (Homo sapiens) AND (HCC OR (hepatocellular carcinoma)) AND (PBMC OR (peripheral blood mononuclear cell) OR (white blood cell) OR (WBC))”. Inclusion criteria were (1) PBMCs or any other white blood cells’ expression file (2) Including both healthy donor cases and HCC cases (3) Datasets of homo sapiens. As a result, two gene expression datasets that compared between PBMC samples from healthy individuals and HCC patients were selected, GSE49515 and GSE58208. We conducted t-tests in each gene expression of each dataset using “Connection Up and Down Regulation Expression Analysis of Microarrays (CU-DREAM) http://pioneer.netserv.chula.ac.th/~achatcha/CU-DREAM/)”,^57^ to evaluate the intersection genes and obtained 187 upregulated genes from both datasets. Then, three genes with highly significant *p*-values (*p* < 0.001) were selected and used to observed gene expressions in our samples.

### Study population

All samples were recruited from King Chulalongkorn Memorial Hospital, Bangkok, Thailand and included 2 cohorts as the following:

Cohort 1: Samples were collected from June 2018 to January 2019 and included 83 HCC cases, 52 healthy donors, 10 with hepatic fibrosis, 10 with non-alcoholic fatty liver.

Cohort 2: Samples were collected from January 2020 to July 2020 and included 70 HCC cases, 24 healthy donors, 10 with hepatic fibrosis, 10 with non-alcoholic fatty liver.

A total of 153 HCC cases, 76 healthy donors, 20 hepatic fibrosis, and 20 non-alcoholic fatty liver participated in this study. Patients with hepatitis viral infection were excluded from this study. HCC staging was recorded according to current BCLC guidelines. All subjects in this study were of Asian descent, further bioinformation is provided in Table 2.

We then used the preliminary results from both GSE 49515 and GSE 58208 to find the appropriate sample size with the following formula:

$$ {\text{n}} = \left[ {\left( {{\text{Z}}_{{\upalpha /2}} + {\text{Z}}_{\upbeta } } \right)^{2} \left( {\upsigma _{{\text{d}}}^{2} } \right)} \right]/(\overline{X}_{{\text{d}}} )^{2} $$

n = sample size.

d = Different of value in each group.

$\overline{X }$_d_ = Different of mean in each group.

σ^2^_d_ = Different of variance in each group.

Z_α/2_ = Standard normal variate for level of significance.

Z_β_ = Standard normal variate for power.

We calculated and found that the sample size for our study was 24.44 samples, confirming that our study has recruited enough samples for the experimentation.

### Blood sampling and PBMC extraction

Two ml of EDTA blood was extracted from all patients. Lymphocyte isolation medium was added to a 15 ml tube and centrifuged at 1600 rpm at 16 °C for 12 min and the plasma was separated. Whole blood (diluted 1:1) with PBS was carefully layered on a tube of lymphocyte separation medium and centrifuged at 2800 rpm for 15 min at 16 °C. The cell interface layer was carefully separated into 1.5 ml tubes and cells were washed with 1 ml PBS for 15 min at 1700 rpm 16 °C and 500 ml PBS for 5 min at 4 °C. The research methodology employed in this project was approved by The Institutional Review Board of the Faculty of Medicine, Chulalongkorn University, Bangkok, Thailand (IRB No. 108/60 and 438/60). All study subjects provided written informed consent.

### RNA extraction

PBMCs were mixed with 1 ml of TRIzol reagent (ThermoFisher Scientific, MA, USA) and incubated at room temperature for 5 min, then 200 μl of chloroform was added and incubated at room temperature for 3 min. Thereafter, it was separated into three phases by centrifugation at 8760 rpm at 4 °C for 15 min. The colorless upper aqueous phase was transferred to a new RNA tube, supplemented with 4 μL of glycogen (20 mg / mL) and 500 μl of 100% isopropanol, incubated for 10 min at room temperature, then centrifuged at 8760 rpm at 4 °C for 15 min. The supernatants of the centrifuged tubes were discarded, and the RNA pellets were washed with 1 ml of 75% ethanol, mixed by vortexing, and centrifuged at 6930 rpm at 4 °C for 5 min. Thereafter, supernatant was discarded again, and RNA pellet was dried by vacuum for 8 min and resuspended with 30 μL of DEPC water. RNA concentration and integrity were confirmed by Nanodrop and bioanalyzer.

### Complementary DNA (cDNA) synthesis

After, the total RNA was extracted from PBMCs using TRIzol reagent (Thermo Scientific) according to the manufacturer’s protocol. Then, cDNA was synthesized using RevertAid First Strand cDNA Synthesis (Thermo Scientific). The process of cDNA synthesis is as follows: thaw, mix and centrifuge the components of the kit then add the template RNA 0.1 ng—5 µg, primer 1 µL, nuclease-free water up to 12 µL, 5X reaction buffer 4 µL, Ribolock RNAse inhibitor 1 µL, 10 mM dNTP mix 2 µL, RevertAid M-MuLV RT 1 µL. After mixing and brief centrifuging, the samples were incubated for 5 min at 25 °C followed by 60 min at 42 °C. Finally, terminate the reaction by heating at 70 °C for 5 min. The product of the first strand cDNA synthesis can be used directly in PCR or qPCR.

### Primer preparation

Primers were designed using Primer3plus^58^ (for FLNA) and Primer Blast^59^ (for CAP1 and CLU). Primers were synthesized by BIONEER. Each primer sequence, melting temperature, and product length are shown in Table 5. Prior to quantitative PCR, conventional PCR and electrophoresis for finding optimal temperature for each primer was conducted.

**Table 5:** Details of forward and reverse primer sequences of three candidated genes used for qRT-PCR analysis.

Gene	Forward	Reverse	Tm	Product length (bp)
CLU	CAGGCCATGGACATCCACTT	GTCATCGTCGCCTTCTCGTA	60.03	78
FLNA	TTTCCGCCAAATGCAGCTTG	ACACCAGTTTGATGCTCTCG	60.32	74
CAP1	GGAACTCTGAGGTGGTCCATTA	ACGGTGCATGTCAGAGGTATG	60.13	108

### Quantitative Real-time PCR (qRT-PCR) analysis

The quantitative PCR contained 10 µl SensiFast (Bioline), 0.8 µl of forward and reverse primers, cDNA(1 µl for FLNA, 0.5 µl for CLU and CAP1), and 7.4 µl distilled water in a total volume of 20 µl. The reactions were carried out on QuantStudio 6 (Thermo Fisher Scientific) according to the manufacturer’s protocol. PCR conditions were as follows: denaturation at 95 °C for 2 min with 45 cycles, annealing at 59 °C, 55 °C, 59 °C for CLU, FLNA, CAP1, respectively for 30 s. Fluorescence signals from the amplified product were detected at the end of the annealing step. Duplications were done on available and unamplified samples. The Ct value was set to 45 if the sample did not show any amplification twice. In this study, the housekeeping gene or the reference gene, that was used is glyceraldehyde 3-phosphate dehydrogenase (GAPDH). We used this gene to test and analyze alongside our interested gene (CLU, FLNA, and CAP1).

The calculation is as follows:

$$ \Delta \Delta {\text{Ct}} = \Delta {\text{Ct }}\left( {{\text{a\,target\,sample}}} \right) - \Delta {\text{Ct}}\left( {{\text{a\,reference\,sample}}} \right) $$

The final result is represented in the folds of change (thus, the equation is in the power of 2 or 2^−ΔΔCt^) of the interested gene expression in the sample against the reference sample^60^.

### Statistical analysis

Box plot, summary of the dataset (including t-test results of Ct mean of each gene), benchmarks (Accuracy, Sensitivity, and Specificity) heatmaps (confusion matrices) and the Receiver Operating Characteristic (ROC) curves were drawn with python 3.9 program with packages (scipy^61^, pandas^62^ and matplotlib^63^). Ct values of each gene were included into the dataset. For the evaluation of performance, the entire dataset was used for the test. The *p*-value cut-off for each test was at < 0.05 for results to be statistically significant.

### Ethical statement

The research methodology employed in this project was approved by The Institutional Review Board of the Faculty of Medicine, Chulalongkorn University, Bangkok, Thailand (IRB No. 108/60 and 438/60). All study subjects provided written informed consent.

## Abbreviations

AFP: Alpha-Fetoprotein
APCs: Antigen Presenting Cells
AP-1: Activator protein 1
cAMP: Cyclic adenosine monophosphate
CAP1: Cyclase Associated Actin Cytoskeleton Regulatory Protein 1
CLU: Clusterin
Ct: Cycle threshold
CTLA-4: Cytotoxic T-Lymphocytes-Associated protein 4
FLNA: Filamin A
GAPDH: Glyceraldehyde 3-phosphate dehydrogenase
GEO: Gene Expression Omnibus
HCC: Hepatocellular Carcinoma
HF: Hepatic Fibrosis
IL-1β: Interleukin 1 beta
IL-2: Interleukin-2
IL-6: Interleukin-6
IL-10: Interleukin-10
MHC: Major Histocompatibility Complex
NAFL: Non-Alcoholic Fatty Liver
NFAT: Nuclear factor of activated T-cells
NF-κB: Nuclear Factor Kappa B
PBMCs: Peripheral Blood Mononuclear Cells
PD-L1: Program death ligand 1
PD-L2: Program death ligand 2
PKA: Protein kinase A
PKC-θ: Protein kinase C-θ
qRT-PCR: Quantitative Real Time – Polymerase Chain Reaction
ROC: Receiver operating characteristic
TCR: T-cell receptor
TGF-β: Transforming Growth factor beta
TNFα: Tumor necrosis factor alpha
WBCs: White Blood Cells
